# Hepatocellular Carcinoma: Optimal Radiological Evaluation before Liver Transplantation

**DOI:** 10.3390/life13122267

**Published:** 2023-11-27

**Authors:** Marco Dioguardi Burgio, Lorenzo Garzelli, Roberto Cannella, Maxime Ronot, Valérie Vilgrain

**Affiliations:** 1 Department of Radiology, Hôpital Beaujon, AP-HP. Nord, 100 Boulevard du Général Leclerc, 92110 Clichy, Francevalerie.vilgrain@aphp.fr (V.V.); 2 Centre de Recherche sur l’Inflammation, UMR1149, Université Paris Cité, 75018 Paris, France; 3 Service d’Imagerie Medicale, Centre Hospitalier de Cayenne, Avenue des Flamboyants, Cayenne 97306, French Guiana; 4 Department of Biomedicine, Neuroscience and Advanced Diagnostics (Bi.N.D.), University Hospital “Paolo Giaccone”, Via del Vespro 129, 90127 Palermo, Italy

**Keywords:** carcinoma, hepatocellular, liver, transplantation, liver

## Abstract

Liver transplantation (LT) is the recommended curative-intent treatment for patients with early or intermediate-stage hepatocellular carcinoma (HCC) who are ineligible for resection. Imaging plays a central role in staging and for selecting the best LT candidates. This review will discuss recent developments in pre-LT imaging assessment, in particular LT eligibility criteria on imaging, the technical requirements and the diagnostic performance of imaging for the pre-LT diagnosis of HCC including the recent Liver Imaging Reporting and Data System (LI-RADS) criteria, the evaluation of the response to locoregional therapy, as well as the non-invasive prediction of HCC aggressiveness and its impact on the outcome of LT. We will also briefly discuss the role of nuclear medicine in the pre-LT evaluation and the emerging role of artificial intelligence models in patients with HCC.

## 1. Introduction

Hepatocellular carcinoma (HCC) is the most common primary liver cancer and mainly occurs in patients with chronic liver disease and cirrhosis. Liver transplantation (LT) is the recommended curative-intent treatment for patients with early or intermediate stage HCC who are ineligible for resection [1]. Indeed, LT has the advantage of simultaneously treating both the tumor and the underlying chronic liver disease.

Because of chronic organ shortages, the accurate selection of candidates for LT is essential. Cross-sectional imaging, including computed tomography (CT) and magnetic resonance imaging (MRI), plays a pivotal role in the diagnosis of HCC, in the selection of patients eligible for LT, and in the assessment of tumor response following locoregional therapy (LRT).

This review discusses the role of the radiological examination, mainly CT and MRI for the staging of patients with HCC before LT, and discusses the prognostic role of imaging in these patients.

## 2. Eligibility Criteria for Liver Transplantation

Because of chronic organ shortages, it is essential to identify patients with the greatest need for, and who will benefit most from LT. In the past three decades, numerous selection criteria have been developed for LT in patients with HCC. The aim of these criteria is to select patients, mainly based on tumor burden, so that survival after LT for HCC is similar to that of patients without HCC (Table 1). The Milan criteria, the first patient selection criteria, were published in 1996 and have become the most extensively validated and widely used criteria in clinical practice (and the only model evaluated in a prospective trial).

According to the Milan criteria, a patient is considered eligible for LT in the presence of a single tumor of 5 cm or less or up to three tumors that are 3 cm or less each, with no macrovascular invasion and no metastases [2]. The imaging method to measure tumor size was not mentioned in that study, and the diagnosis was based on either biopsy or a serum alpha-fetoprotein > 300 ng/mL. Since the original publication, the Milan criteria have been adopted in LT centers worldwide and incorporated into staging systems (American Liver Tumor Study Group Modified Tumor-Node-Metastasis, Barcelona Clinic Liver Cancer [3]), and they are commonly used in subgroup analysis in RCT trials [4,5].

The Organ Procurement and Transplantation Network (OPTN) criteria were developed by an expert panel [6,7] to improve the radiological evaluation of HCC before LT and because of the significant discrepancies between radiological and pathological staging. This classification system is a direct application of the Milan Criteria (OPTN Class 5A and 5B are associated with HCC not exceeding 5 cm).

After several studies reported nearly equivalent survival between Milan and subgroups of patients transplanted outside of the Milan criteria, this model was challenged as being too strict (in particular due to their dichotomous yes/no design). Extended Milan criteria were then developed and applied in different LT centers, in particular in 2001, with the University of California San Francisco (UCSF) criteria [8]. The UCSF criteria consider patients with a solitary tumor of no more than 6.5 cm, or up to three nodules with the largest lesion being no more than 4.5 cm and total tumor diameter being no more than 8 cm, to be eligible for LT. Tumor size was determined either via ultrasound (US), CT, or MRI, and it is interesting to note that there were significant discrepancies in accuracy for the three imaging modalities. In 2009, the first version of the so-called Metroticket [9] criteria was developed based on a retrospective international survey analysis to improve the prediction of survival after LT. Five-year survival in patients outside the Milan criteria without microvascular invasion and who met a new criterion called “Up-to-Seven” (with the sum of the size of the tumors (in cm) and the number of tumors not exceeding 7) was 71% (which was similar to the 4-year overall survival of 75% using the Milan criteria). However, in this study, pathology of the explanted liver and not imaging was the reference for tumor measurement. Because increased AFP levels are related to the microvascular invasion of HCC, in 2012, a French retrospective multicenter analysis designed a model that added AFP to tumor burden. This model improved the predictive value of the Milan criteria for survival [10] and once again, pathology on the explanted liver was the reference for tumor measurement.

Metroticket 2.0 was developed in 2018 using a more practical, tailored approach to preoperative tumor measurement [11]. This predictive regression model of death following LT for HCC, based on AFP levels and tumor features (size and number), outperformed the Milan, UCSF, Up-to-Seven, and AFP-French criteria and has now been endorsed by both the European Liver and Intestine Transplant Association and the International Liver Transplantation Society.

The Liver Imaging Reporting and Data System (LI-RADS) was recently developed [12] and has been included in the American Association for the Study of Liver Disease (AASLD) guidelines for the diagnosis and treatment of HCC [13].

The evidence on LI-RADS and its value in pre-LT staging is limited, and the main question is whether to include LR-3, LR-4, or LR-M in the determination of the tumor burden. A retrospective single-center study showed that the diagnostic accuracy of the Metroticket 2.0 calculator was reduced when LR-3 and LR-4 nodules were excluded [14], while another recent study showed that the accuracy of LI-RADS was similar for the assessment of LT eligibility based on the Milan criteria when LR-4 and LR-M were included in addition to LR-5 observations [15].

**Table 1:** Summary of the available eligibility criteria for liver transplantation in hepatocellular carcinoma.

Eligibility Criteria for LT	Criteria	Design	Reference for Tumor Measurement	HCC Diagnosis	Population Size within Criteria	Survival
**Milan** *Mazzaferro V, 1996* [2]	Single tumor ≤ 5 cm 3 tumors all ≤ 3 cm	Prospective Monocentric	Not mentioned	Biopsy or AFP > 300 ng/mL	48	4-year OS: 75%
**UCSF** *Yao FY, 2001* [8]	Single tumor ≤ 6.5 cm 3 tumors all ≤ 4.5 cm Total tumor size ≤ 8 cm	Retrospective Monocentric	CT (42%), MRI (20%), US (38%)	Pathology on liver explants	70	5-year OS: 75%
**Up-to-Seven** (Metroticket 1.0) *Mazzaferro V, 2009* [9]	Sum of the size of all tumors + number of tumors ≤ 7 No vascular invasion	Retrospective Multicentric (Survey form)	Pathology on liver explants	Pathology on liver explants	283	5-year OS: 75%
**French-AFP cohort** *Duvoux C, 2012* [10]	Score ≤ 2 (model including largest diameter, AFP, number of tumors)	Retrospective Multicentric (France) Validation cohort (France)	Pathology on liver explants	Pathology on liver explants	791	5-year OS: 67%
**Metroticket 2.0** *Mazzaferro V, 2018* [11]	Up-to-Seven + AFP <200 ng/mL Up-to-Five + AFP 200–400 ng/mL Up-to-Four + AFP < 400–1000 ng/mL	Retrospective Multicentric (Italy) Validation cohort (China)	CT, MRI	Biopsy or imaging (EASL/AASLD guidelines)	1055	5-year OS: 81%

AFP: alpha-Fetoprotein; AASLD: American Association for the Study of Liver Diseases; EASL: European Associations for the Study of the Liver; OS: Overall Survival, UCSF: University of California San Francisco.

## 3. CT and MRI Technical Requirements and Protocols

Cross sectional imaging including both multidetector CT scan and MRI is used for pre-LT tumor staging. Contrast-enhanced CT provides a whole-body evaluation. This technique is used to assess tumor burden and exclude certain important contra-indications to LT [16], mainly the presence of metastatic disease, other extrahepatic malignancies, and macrovascular tumoral invasion. Moreover, contrast-enhanced CT provides an accurate evaluation of liver volume and vasculature which is essential for an optimal surgical strategy.

Liver MRI is usually performed as a second line technique or in combination with CT. Besides its added value in the detection and characterization of focal liver lesions, liver MRI provides a multiparametric evaluation of the liver, and additional data that cannot be accurately obtained from enhanced CT. This includes an evaluation of the biliary tree anatomy via MR cholangiopancreatography, and quantification of hepatic fat or fibrosis as well as liver function using hepatobiliary contrast agents (HBA).

The recommended technical requirements for CT and MRI to maximize tumor detection and characterization are reported in the LI-RADS v2018 criteria [17].

A multidetector CT with at least eight detector rows should be used. The acquisition phase should include one late arterial phase (with bolus tracking technique or 35″ delay) after contrast administration, a portal venous phase (70–80″) and a delayed phase (three minutes). CT should cover cerebral, cervicothoracic, and abdominal images.

A 1.5 T scan with a phased-array multichannel torso coil should be used for MRI. The protocol includes an unenhanced in-phase and an opposed-phase gradient echo T1-weighted sequence, a T2-weighted sequence, and also multiphase T1-weighted images including a precontrast acquisition, late arterial phase, and portal venous phase images. If extracellular contrast agents or gadobenate dimeglumine are used, a delayed phase is acquired at three minutes, while a hepatobiliary phase (HBP) acquisition using HBA is strongly suggested 1 to 3 h after contrast administration. If gadoxetate disodium enhanced-MRI is performed, a transitional phase (2 to 5 min) is followed by the HBP (15–20 min).

### The Added Value of the Hepatobiliary Phase

A HBP acquisition can be obtained with MR HBA administration, either gadobenate dimeglumine or gadoxetate disodium. After a vascular distribution which is similar to that of traditional extracellular MR contrast agents, these molecules are selectively taken up by the hepatocytes and excreted into the biliary system. Unlike normal hepatocytes, most HCCs lose the ability to take up these molecules [18]. Although HBP acquisitions are not necessary to obtain a diagnosis of HCC in high-risk patients, they provide important pre-LT information.

In addition to better detection of nodules, in particular with hepatic arterial phase hypoenhancement and hypointensity on HBP acquisitions, HBP images have prognostic value in patients with HCC. Marked hepatobiliary phase hypointensity is correlated with HCC aggressiveness [19], and peritumoral hypointensity is associated with the presence of microvascular invasion (MVI) [20,21]. A recent meta-analysis has also reported an increased risk of tumor recurrence after resection or LT in the presence of hypointense HCC on HBP acquisitions [19].

## 4. The Performance of CT and MRI for the Diagnosis of HCC before Liver Transplantation

Contrast-enhanced CT and MRI are the recommended imaging modalities for the noninvasive diagnosis of HCC before LT. Both techniques are highly specific for the noninvasive diagnosis of HCC in high-risk patients. However, MRI has been shown to be more sensitive for the diagnosis of HCC with better detection of lesions in LT candidates [22]. One retrospective study by Bae et al. that included 136 patients who underwent contrast-enhanced CT before LT reported a sensitivity of 57–69% and a specificity of 82–87% for the diagnosis of HCC using the LI-RADS criteria, with an overall accuracy for LT eligibility of 85–93% [15]. A study by Seeman et al. [23] reported that contrast-enhanced CT, extracellular contrast MRI, and gadoxetate disodium MRI had sensitivities of 59.5%, 78.5%, and 76.8%, respectively, for the diagnosis of HCC in patients who underwent LT and a specificity of 97.0%, 99.1%, and 91.2%, respectively.

The combination of imaging features for a confirmed HCC diagnosis differs among guidelines. The European Association for the Study of the Liver (EASL) criteria state that a non-invasive diagnosis of HCC can be made in patients with cirrhosis in the presence of lesions ≥ 10 mm with arterial phase hyperenhancement (APHE) and washout in the portal venous or delayed phases on CT or MRI with extracellular contrast agents or gadobenate dimeglumine, or washout in the portal venous phase only on MRI with gadoxetate disodium [24]. The LI-RADS v2018 criteria were endorsed in the AASLD practice guidelines for the diagnosis, staging and management of HCC and may be applied to high-risk patients including LT candidates with cirrhosis, chronic hepatitis B, and a prior or current history of HCC [12,25]. A definite diagnosis of HCC (LR-5) is based on the combination of observed size (at least 10 mm), nonrim APHE, and other major imaging features including non-peripheral washout (evaluated in the portal venous phase only with gadoxetate disodium), an enhancing capsule, and threshold growth [12]. It is interesting to note that LR-5 criteria are in agreement with the OPTN/UNOS criteria except for the following observations: 10–19 mm with nonrim APHE and nonperipheral washout [7,26].

A definite diagnosis of HCC in the recently updated Korean Liver Cancer Association-National Cancer Center (KLCA-NCC) guidelines includes nodules ≥ 10 mm with APHE and washout on the portal venous or delayed phase, or hypointensity on hepatobiliary phases in lesions with no marked T2 hyperintensity or targetoid appearance [27]. In the Asian Pacific Association for the Study of the Liver (APASL) guidelines, the diagnosis of HCC is confirmed in lesions with APHE and portal venous washout or hepatobiliary phase hypointensity rather than washout, regardless of lesion size [28]. Both the KLCA-NCC and APASL guidelines consider patients with cirrhosis and chronic viral hepatis B or C to be at high risk of HCC even in the absence of cirrhosis [27,28].

Very few studies have compared the sensitivity and specificity of existing imaging guidelines for the pre-LT assessment (Table 2) [29,30,31,32]. Existing studies all report that KLCA-NCC and APASL guidelines are more sensitive for the diagnosis of HCC in patients examined with gadoxetate disodium MRI, while EASL and LI-RADS guidelines are more specific. Differences among guidelines are probably due to the inclusion of HBP hypointensity as a major feature for the diagnosis of HCC in KLCA-NCC and APASL criteria, resulting in false positive diagnoses of HCC in patients with dysplastic nodules, small intrahepatic cholangiocarcinoma, or combined hepatocellular-cholangiocarcinoma, which may have nonrim APHE and HBP hypointensity [33].

**Figure 1:**
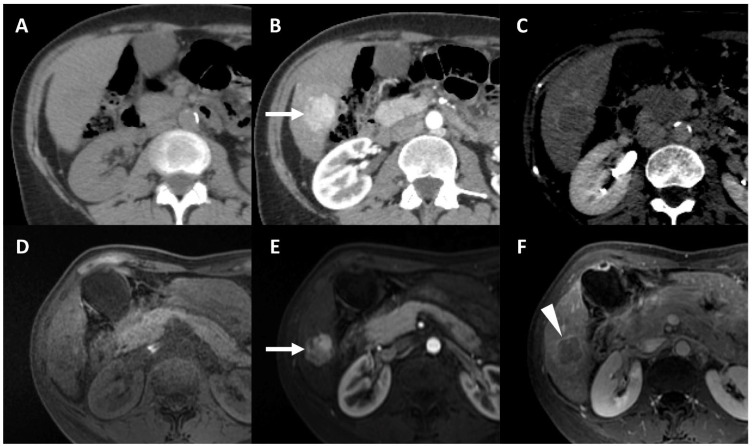
Typical appearance of hepatocellular carcinoma (HCC) on both contrast enhanced CT (**A**–**C**) and MRI (**D**–**F**) in the segment VI of the liver in a patient with hepatitis C-related liver cirrhosis. At unenhanced CT (**A**), the lesion is isoattenuating, while it is slightly hypointense on unenhanced T1 fat-saturated MR image (**D**), compared to the background liver. The lesion shows nonrim arterial phase hyperenhancement on both CT ((**B**) arrow) and MRI ((**E**), arrow) and washout on delayed venous phases on both CT (**C**) and MRI (**F**). Note the enhancing capsule which is best depicted on delayed phase at MRI ((**F**) arrowhead).

Although LI-RADS observations categorized as LR-3 (intermediate probability of malignancy) or LR-4 (probably HCC) are not considered to be HCC lesions for the assessment of LT eligibility, they have a 31–38% and 64–74% probability of being HCC, respectively [34,35]. Combining LR-5 and LR-4 categories can increase the sensitivity for the diagnosis of HCC in LT candidates; however, a slight decrease in specificity has also been observed [31]. A study by Piñero et al. [36] reported no significant difference in the percentage of HCC between LR-5 and LR-4 observations, but that study did not include patients with non-HCC malignancies on liver explants. The categorization of LR-4 observations in LT candidates should be managed in multidisciplinary meetings, and a biopsy may also be included to confirm the diagnosis of HCC. Observations categorized as LR-M (probably or definitively malignant but not HCC-specific) should not be considered eligible for LT unless the diagnosis of HCC is confirmed by biopsy [37]. Indeed, most LR-M correspond to non-HCC malignancies such as intrahepatic cholangiocarcinoma and combined hepatocellular-cholangiocarcinoma, which have a poor prognosis following LT [38,39]. An example of the typical appearance of HCC on CT and MRI is provided in Figure 1.

## 5. Evaluation of Tumoral Response after Local Regional Therapy

There are two goals for local regional therapy (LRT) in patients with cirrhosis and HCC waiting for LT: first, to achieve local control of the disease and prevent patients from dropping off the waiting list (bridging); and second, to downstage patients who are outside LT criteria to make them eligible for transplantation.

LRT is a well-established technique that can reduce postoperative tumor recurrence and improve overall survival after LT [40]. This is probably due to local tumor control and the selection of patients with a more favorable tumor biology.

LRT mainly includes transarterial chemoembolization (TACE) and percutaneous thermal ablation with radiofrequency or microwaves [41]. More rarely, transarterial radioembolization (TARE), cryoablation or non-thermal ablation techniques such as irreversible electroporation or external radiation beam therapy may be performed before LT.

The role of post-LRT imaging is to assess tumor response to select the best candidates for LT. Although the Response Evaluation Criteria in Solid Tumors (RECIST) 1.1 [42] are commonly used in oncology to assess the radiological response after chemotherapy, they are not suitable for assessing the response of HCC after LRT because these criteria are based on tumor diameter reduction and underestimate tumor necrosis [43].

Thus, more appropriate criteria have been developed. The modified RECIST (mRECIST) [44], which assess tumor response by evaluating the viable (APHE) portion of the target lesion(s) (up to two for each organ) are the most widely used criteria. Similarly to RECIST 1.1, mRECIST classifies tumor response into four categories: complete response (no APHE in any target lesion), partial response (≥30% reduction in the sum of diameters of enhancing parts of target lesions), progressive disease (≥20% increase in the sum of diameters of enhancing parts of target lesions), and stable disease (non-classifiable as neither partial nor progressive disease). mRECIST criteria have been shown to differentiate responders and non-responders compared to pathological examination [45]. Moreover, a complete response according to mRECIST has been found to be associated with increased overall survival in patients initially outside the Milan Criteria [46], while failure to respond to LRT was associated with an increased risk of dropout from the waiting list as well as recurrence after LT [47]. The recent incorporation of mRECIST into Metroticket 2.0 has improved the predictive accuracy of tumor-related deaths after LT. In particular the 5-year HCC-related death rates were 3.1%, 9.6%, and 13.4% in patients classified with a complete response, partial/stable disease, or progressive disease, respectively [48].

The Liver Imaging Reporting and Data System (LI-RADS) was developed to standardize the terminology, technique, interpretation, reporting, and data collection of liver imaging [12]. The most recent LI-RADS v2018 provides a treatment response algorithm for patients treated with LRT based on the visual assessment of tumor viability. Tumors are defined as nodular, mass-like, or thick, with irregular tissue in or along the treated lesion showing APHE or washout appearance, or enhancement similar to that observed before LRT. Lesions are classified as nonviable, equivocal, or viable after LRT.

**Figure 2:**
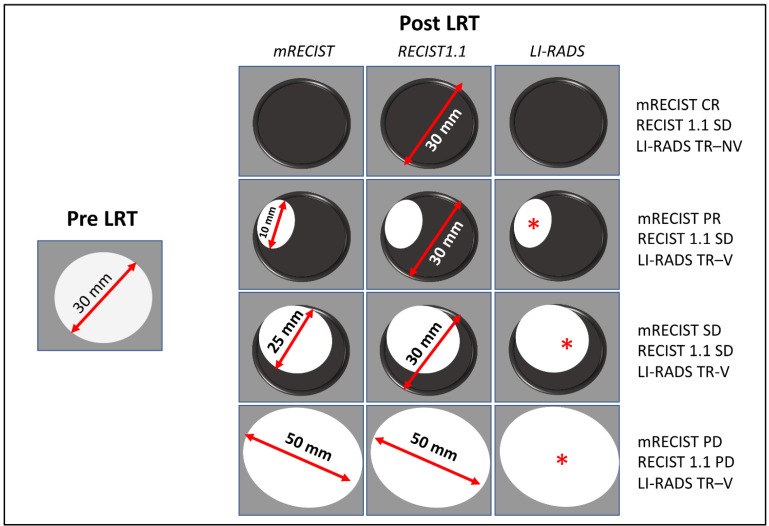
Schematic application of RECIST 1.1, mRECIST, and LI-RADS criteria for assessing response after local regional therapy (LRT) in hepatocellular carcinoma. After LRT, four examples are provided and represented in each line. The arterial phase hyperenhancing portion of the tumor is represented in white. Please note the discordance among response classification between RECIST 1.1, which takes into account the whole tumor burden, compared to mRECIST and LI-RADS criteria. CR: complete response; PD: progressive disease; SD: stable disease; TR-NV: treated non-viable; TR-V: treated viable. *: viable tumor.

When the LI-RADS treatment algorithm is applied to CT and MRI, both techniques are found to be highly specific (94% and 95%, respectively) in evaluating tumor viability after LRT, while the sensitivity of MRI is slightly higher with a hepatobiliary contrast agent (52% vs. 42%) [49]. A meta-analysis including six studies with 393 patients and 534 observations based on resection or LT as a reference, reported a pooled sensitivity for the LI-RADS treatment algorithm of 0.56 and a specificity of 0.91 for the detection of incomplete necrosis after LRT [50]. Another recent meta-analysis including five studies with 430 patients with 631 treated observations also showed that the LI-RADS algorithm was more specific than mRECIST for detecting pathologically viable HCC after LRT (pooled specificity 93% (95% CI, 88%–96%) vs. 86% (95% CI, 72%–94%), respectively), with no significant difference in sensitivity [51]. An example of the application of RECIST 1.1, mRECIST, and LI-RADS is provided in Figure 2.

The post-LRT imaging assessment is usually performed 3 to 6 weeks after LRT. There is no standardized follow-up protocol to assess response after LRT. Both CT and MRI may be used depending on patient characteristics, type of treatment, pre-treatment localization and lesion visibility, local availability, and this protocol should be repeated every three months until LT.

### 5.1. Evaluation following Percutaneous Thermal Ablation and TACE

Percutaneous thermal ablation is performed using radiofrequency or microwaves devices. Heat induces cell death and creates a histological zone called “coagulative necrosis” in the treated portion of the liver parenchyma. This usually is seen on follow-up imaging as a hypoattenuating area (ablation zone) on CT and a spontaneously hyperintense area on T1-weighted MRI.

**Figure 3:**
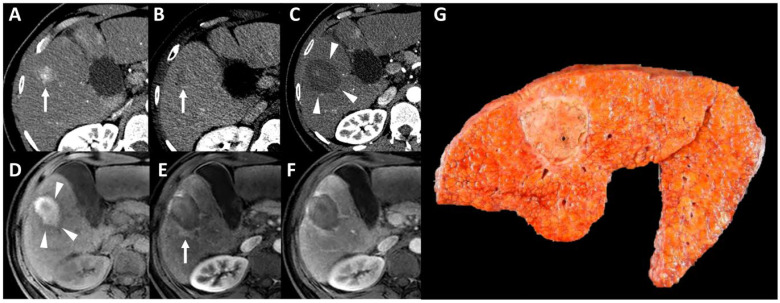
Example of imaging evaluation after thermal ablation as bridging treatment in a 55-year-old patient with hepatitis C-related cirrhosis complicated by hepatocellular carcinoma (HCC). Pretreatment contrast-enhanced CT (**A**,**B**) shows a 2 cm HCC in the segment V of the liver ((**A**,**B**) arrows). After the microwave ablation (**C**–**F**) contrast-enhanced CT obtained during arterial phase shows the appearance of an hypoattenuating ablation zone (**C**, arrowheads) and the disappearance of the arterial phase hyperenhancement of the lesion previously shown in (**A**). Unenhanced (**D**) and gadolinium-enhanced (**E**,**F**) fat-saturated T1-weighted MRI images show a hyperintense appearance of the ablation zone ((**D**) arrowheads) and the disappearance of the arterial phase hyperenhancement. Please note the presence of a peripheral enhancement surrounding the ablation zone ((**E**) arrow), with no washout on portal venous phase (**F**), which is consistent with a perfusion alteration. Note that the ablation zone surrounds the treated lesion. Histopathological analysis of the liver specimen after transplantation (**G**) confirmed the complete necrosis of the lesion.

It is important to confirm that the entire lesion is within the ablation zone, ideally with a safety margin of at least 5 mm between the tumor and the ablation margins, and that there is no residual tumor enhancement (i.e., APHE) after contrast administration, suggesting viability on either CT or MRI—Figure 3. Peripheral enhancement surrounding the ablation zone is commonly seen due to hyperarterialization of the area. This fades over time. Lack of washout, a non-nodular appearance, lack of diffusion restriction, and possible isointensity on MRI HBP when a HBA is used help differentiate it from a residual viable tumor.

The imaging characteristics following TACE largely depend upon the technique. The treated lesion has a necrotic appearance when drug-eluting beads (non-absorbable embolic microspheres loaded with cytotoxic agents) are used and the characteristic APHE is lost in case of a response to treatment. However, the most commonly used technique is the injection of an ethyl ester of iodized fatty acids of poppy seed oil (Lipiodol Guerbet, Aulnay-sous-Bois, France) mixed with a cytotoxic agent (usually doxorubicin or cis-platinum, and more recently idarubicin) [52,53], followed by the administration of an embolic agent (mainly a gelatin sponge).

Lipiodol is characterized by high attenuation on CT. Because this can mask the presence of residual viable tumor by reducing the visibility of enhanced residual components, some authors suggest performing MRI to assess tumor response after conventional TACE [54].

Nevertheless, the presence of lipiodol deposits on CT provides important prognostic features.

**Figure 4:**
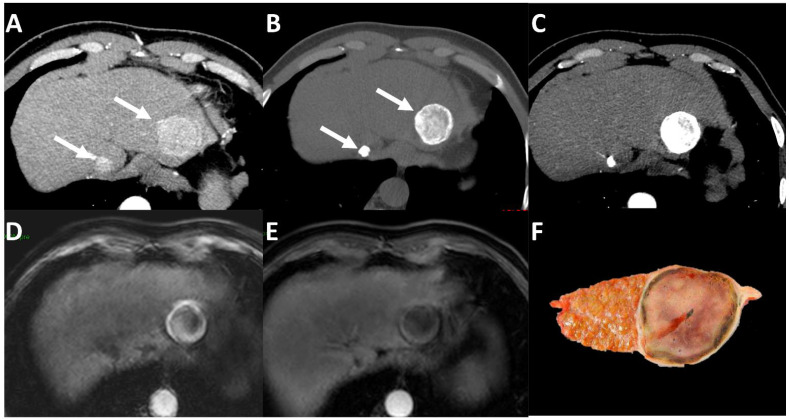
Example of imaging evaluation after transarterial chemoembolization in a 48-year-old patient with hepatitis C-related cirrhosis complicated by two hepatocellular carcinomas (HCC). Pretreatment contrast-enhanced CT (**A**) obtained during hepatic arterial phase shows two hepatocellular carcinomas ((**A**) arrows) with nonrim arterial phase hyperenhancement. The lesion of the left liver lobe is 4 cm, while the lesion in the segment I is 1 cm. After transarterial conventional chemoembolization, both lesions show complete Lipiodol uptake at unenhanced CT ((**B**) arrows) and the disappearance of the arterial phase hyperenhancement (**C**). Unenhanced (**D**) and gadolinium-enhanced (**E**) fat-saturated T1-weighted MRI images show an hyperintense appearance of the larger nodule (**D**) and an hypointense appearance of the smaller and confirm lack of arterial phase hyperenhancement for both lesions (**E**). Histopathological analysis of the liver specimen after transplantation (**F**) confirmed the complete necrosis of the lesions.

Indeed, the presence of complete embolization with a lipiodol deposition throughout the lesion, combined with a complete response according to mRECIST criteria (i.e., disappearance of APHE appearance) is associated with more pathological necrosis, compared to a lesion with a complete response but with incomplete lipiodol deposition [43]. Moreover, the risk of local progression is increased in lesions classified as having a complete response according to mRECIST but with an incomplete lipiodol deposition [55]. Indeed, the lipiodol deposition pattern on CT should be considered an important prognostic feature in the imaging assessment of HCC following conventional TACE—Figure 4.

### 5.2. Evaluation after TARE

Trans-arterial radioembolization (TARE) involves the arterial injection of yttrium-90 (^90^Y) or holmium-165 (^165^Ho) microspheres. Although TARE is not usually used for bridging or downstaging LRT for HCC before LT, certain data suggest that it is safe and effective for this indication [56].

**Figure 5:**
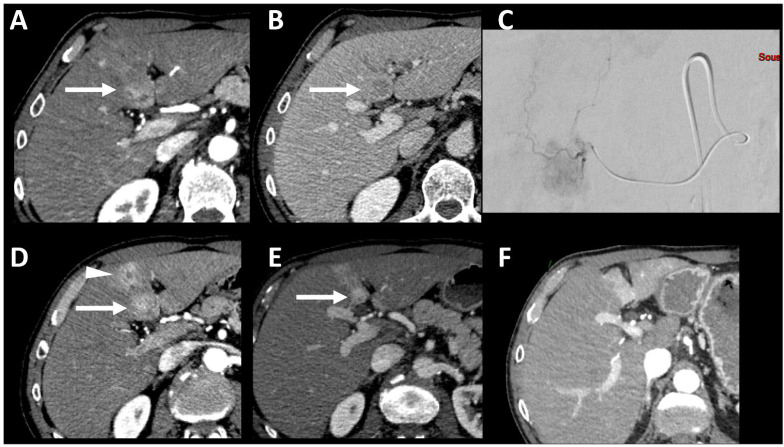
Imaging follow-up appearance after TARE for a hepatocellular carcinoma of the segment IV of the liver in a 61-year-old male. Pretreatment contrast-enhanced CT images (**A**,**B**) obtained during hepatic arterial phase (**A**) and portal venous phase (**B**), show a hepatocellular carcinoma in the segment IV (arrows). Digital subtraction angiography image (**C**) shows selective catheterization and injection of ^90^Yttrium microspheres in the segmental branch of the segment IV. 3-, 6- and 12-month follow-up CT images ((**D**–**F**), respectively) obtained during arterial phase show a slow progressive reduction in the lesion size and a persisting arterial phase hyperenhancement at 3 and 6 months ((**D**,**E**) arrows). Note the appearance of a peritumoral arterial enhancement 3 months after treatment ((**D**) arrowhead), which should not be misinterpreted with tumor progression, as well as the progressive atrophy of the segment IV, which is almost complete at 12 months (**F**).

Changes induced in the tumor and surrounding liver are different from those with TACE. Unlike TACE, TARE microspheres have no or a minimal embolic effect, and thus, tumor necrosis and shrinkage following TARE is often slower and more delayed. Persistent nodular or diffuse APHE is commonly seen in the first months after treatment even responding tumors [57]. Moreover, peritumoral APHE and parenchymal enhancement often appear after TARE within the treated volume [58]. The application of mRECIST criteria can be challenging following TARE due to the heterogeneous appearance of the tumor and the persistent APHE even in responding tumors. Changes in size from pre-TARE imaging are the most important feature to assess tumor response, and the disappearance of ancillary features that favor malignancy such as diffusion restriction on MRI can also help [57]. Overall, regular follow-up for several months is needed to clearly assess tumor response after TARE. An example of imaging of HCC following TARE treatment is provided in Figure 5.

## 6. Prediction of HCC Aggressiveness and the Impact on LT Outcome

### 6.1. Features of HCC Aggressiveness

Tumor biology and aggressiveness have been shown to directly influence the post-LT risk of tumor recurrence. One of the most important prognostic factors is the extent of local tumor invasion [59,60]. While macro-vascular invasion is often visualized on imaging and is a strict contraindication to LT, the identification of microvascular invasion (MVI) is still a challenge both on imaging and pathologically. Other reported negative histoprognostic factors include high tumor grade (i.e., poor differentiation) [61], vessel encapsulated tumor clusters (VETC) [62,63] and tumor subtype [64]. VETC correspond to tumors encapsulating cells that enter the blood stream to escape immune attacks and apoptosis. VETC have been associated with metastatic dissemination of HCC, high serum alpha-fetoprotein (AFP) levels, larger size, poorer grade, a macrotrabecular pattern, less inflammatory infiltration, and frequent MVI [62]. The World Health Organization (WHO) recommends classifying HCCs into eight specific subtypes based on morphological patterns and molecular features [65]. Some of these subtypes belong to the “proliferative class” that is enriched in TP53 gene mutations and FGF19 or CCND1 amplifications [66,67]. Macrotrabecular-massive HCCs (MTM-HCC), which are the most frequent subtype, represent 5–15% of HCCs, have a very poor prognosis, and are highly invasive with greater metastatic spread [68,69].

### 6.2. Prognostic Value of Imaging

In most patients, the histopathological characteristics of HCC can only be assessed via biopsy before LT, which is prone to sampling errors, or after surgery when LT is considered to be a salvage treatment. However, imaging can also help assess tumor biology [70]. Numerous studies have focused on imaging features that are predictive of the different pathological tumor characteristics.

In one retrospective study, Taouli et al. analyzed 39 HCCs and found positive associations between infiltrative pattern, mosaic appearance, the presence of venous invasion, and large size with aggressive genotypes [71].

Larger size, peritumoral APHE, a disrupted capsule, low apparent diffusion coefficient values, and HBP hypointensity were correlated with poorer tumor differentiation. Studies also suggest that substantial necrosis, low ADC, and larger size may indicate macrotrabecular-massive HCC [72,73]. Feng et al. showed that the MTM-HCC and VETC pattern share common imaging features [74].

### 6.3. Imaging of Microvascular Invasion

The presence of vascular invasion has been known for many years to be a major prognostic factor. Macrovascular invasion is defined by LI-RADS as clearly enhancing soft tissue in vein (portal or venous). The sensitivity of the corresponding LI-RADS tumor in vein (TIV) category is only moderate, while the specificity is excellent for the diagnosis of macrovascular invasion. In a retrospective study including 1322 patients with (*n* = 101) or without (*n* = 1221) TIV at pathology, the sensitivity and specificity of TIV on imaging was 62–64% and 99% on CT and MRI, respectively [75].

The detection of MVI on imaging is more difficult and is mainly based on the analysis of the parenchyma surrounding the tumor. Because the rate of MVI increases with the tumor size (present in 25% and 63% of HCCs < 2 cm and >6.5 cm, respectively) [76,77], nearly all large tumors have MVI. This question is therefore crucial in patients with a small tumor burden, who are typically candidates for LT. The imaging features associated with MVI include nonsmooth tumor margin [78], incomplete tumor capsule [79], low ADC, large size, peritumoral APHE, and peritumoral HBP hypointensity [80]. Peritumoral hypointensity on HBP is the MRI feature that is most suggestive of MVI and can be useful to confirm MVI [21]. Numerous prognostic nomograms or feature clusters have been proposed to predict MVI [81,82,83]. Unfortunately, the interobserver variability of these features is significant, even for experienced readers [84]. A few studies have used more quantitative approaches to define radiomic signatures. Xu et al. showed that the predictive performance of radiomic features on CT were good to predict MVI and clinical outcomes. However, radiomics had no added value compared to features assessed by the radiologist [85]. A recent meta-analysis on the role of radiomics in HCC showed that it was promising but stressed the need for standardization and external validation before it could be used in clinical practice [86].

### 6.4. Imaging Features with Positive Prognostic Value

Not all imaging features are associated with a poor prognosis. Intratumoral fat is more frequently present in early and well-differentiated HCC. Steatohepatitic HCCs—one of the WHO subtypes—also contain fat and belong to the “nonproliferative class” of tumors. They are smaller, with rare MVI and metastases [87]. Cannella et al. showed that fat was significantly more frequent in steatohepatitic HCCs than in other subtypes [73]. However, the presence of fat is not reliable enough to predict the SH-HCC subtype, because this feature was also observed in 8–23% and 22–31% of not-otherwise specified HCCs (i.e., classical HCC) on CT and MRI, respectively, as well as 5–15% and 14–50% of MTM-HCCs, respectively [73]. Iso- to hyperintensity on HBP has also been associated with a better prognosis, possibly related to the upregulation of OATP1B3 by activating mutations in the CTNNB-1 gene, which encodes for β-catenin [88]. These β-catenin–mutated HCCs are well-differentiated tumors with low AFP, less frequent MVI, and a favorable prognosis. Finally, tumor encapsulation, defined as the presence of a fibrous sheath around the tumor on gross inspection, is a good prognostic factor [79].

## 7. The Role of Positron Emission Tomography before Liver Transplantation

^18^F-FDG PET-CT is not routinely used in HCC patients because HCCs have less FDG uptake, resulting in a low sensitivity (50–65%) for the detection of these tumors [89].

Nevertheless, some studies have suggested the potential predictive role of ^18^F-FDG PET for tumor recurrence and aggressiveness, including MVI or tumor differentiation [89,90] after LT for HCC. Thus, some teams systematically combine whole body PET/CT to CT and MRI for HCC staging.

Nevertheless, the results in the literature are discordant. For example, the sensitivities for MVI and tumor differentiation are reported to range from 54% to 87% and from 11% to 85%, respectively [91]. Increased SUV_max_ on PET/CT could be used as an additional predictive marker for patient outcome. One review has reported a disease-free survival rate of approximately 40–50% three years after LT in patients with positive PET/CT compared to approximately 90% in patients with negative PET/CT before LT [92]. However, the most recent AASLD practice guidelines for the prevention, diagnosis, and treatment of HCC do not recommend the use of PET-CT [92].

## 8. Future Directions: The Emerging Role of Artificial Intelligence

In the past few years, numerous artificial intelligence (AI) approaches have been developed with various machine-learning or deep-learning methods in all fields of medicine, in particular radiology.

Radiological images are an inexhaustible source of data that can be processed and combined with information from other areas of medicine, such as demographic, laboratory, or histopathological data.

The growth of these AI approaches could significantly modify LT patient management, from donor–recipient matching, to the prediction of short- (i.e., risk prediction of post-LT graft failure) and long-term outcomes (i.e., risk prediction of HCC recurrence) [93]. Indeed, several AI models have been developed, mainly using CT or MRI images, to improve detection and predict aggressiveness, such as MVI or grading and the molecular evaluation of HCCs [94].

Despite the growing number of publications describing the potential role of these AI approaches in patients with HCC, no radical changes have occurred in clinical practice in recent years as a result. This is mainly due to the significant variability in and lack of reproducibility of much of the published data, which are mainly based on non-prospective single-center cohorts. Indeed, there is a risk of developing models that cannot be extrapolated on a large scale, and thus validated. For example, our team found that the performance of CT radiomics prediction of MVI in HCC was poor, and more importantly that the results varied significantly by randomly modifying the selection of the patients in the training cohort of the model [95].

Thus, the construction of AI models and approaches must be developed in association with accurately standardized data, with large-scale assessments including multicenter and prospective cohorts [96], as well as a published descriptions of standardized methodologies [97].

Despite these limitations, AI models will most probably become additional tools in the coming years to improve therapeutic decision making and patient selection for LT in patients with HCC.

## 9. Tips and Tricks for Daily Practice

This list of tips is provided to synthetize the reported evidence and to give practical recommendations:

- Accurate staging of HCC is of paramount importance in patients with HCC prior to LT.
- CT and MRI should be performed as close as possible to the date of LT to avoid missing any tumor appearance or progression that may potentially affect the clinical outcome of patients.
- The technical standard of CT and MRI should be consistent with LI-RADS guidelines and the use of non-invasive diagnostic criteria for HCC should be adapted to clinical needs; in particular, an increase in diagnostic specificity should be achieved considering the organ shortage.
- Use of the standardized LI-RADS lexicon is recommended.
- Particular attention should be paid to cases with discrepancies between AFP levels and radiological staging and to imaging features that may predict the aggressiveness of HCC (i.e., features associated with MVI, features associated with tumor subtypes, HBP appearance). Although these features are not currently used to select patients for transplantation, it is likely that more detailed evaluation of these features may lead to better optimization of patient selection in the future.
- Knowledge of common radiological appearance and physiological modifications after LRT is necessary to avoid misinterpretation of tumor progression and to correctly assess tumoral response.
- Lipdiodol deposition pattern should be used on CT as a prognostic marker of LRT response.
- For imaging evaluation of HCC after LRT, the RECIST criteria should not be applied as they underestimate pathological response, and the mRECIST or LIRADS algorithm should be applied in this case.

## 10. Conclusions

Imaging plays a central role in the evaluation of patients with HCC prior to LT. In addition to tumor staging and response to LRT, several imaging features can be used as biomarkers to select patients who can best benefit from LT.

## Figures and Tables

**Table 2:** Studies comparing different HCC guidelines in liver transplant setting using the histopathological analysis of the explanted liver as reference standard.

		EASL		AASLD/LI-RADS		KLCA-NCC		APASL	
Study	Modality	Sen (%)	Spec (%)	Sen (%)	Spec (%)	Sen (%)	Spec (%)	Sen (%)	Spec (%)
Clarke et al. [29]	EOB-MRI	44	86	45	89	-	-	64	81
Jeon et al. [30]	EOB-MRI	38.8	92.1	34.5	97.4	65.5	92.1	75.9	78.9
Odedra et al. [31]	CT EOB-MRI	13.6 26.2	100 100	25.2 29.1	100 100	25.2 45.9	100 91.7	31.1 63.1	100 100
Seo et al. [32]	CT	50.0	99.4	40.4	99.4	50.0	99.4	-	-

Note. Percentages are reported for all lesions included in the study. In presence of multiple readers, the sensitivity and specificity of the most experience reader is provided. In AASLD/LI-RADS sensitivity and specificity are reported for LR-5. EOB-MRI: gadoxetate disodium enhanced magnetic resonance imaging.

## Data Availability

No new data were created or analyzed in this study. Data sharing is not applicable to this article.

## Abbreviations

AASLD	American Association for the study of Liver Diseases
APASL	Asian Pacific Association for the Study of the Liver
APHE	Arterial phase hyperenhancement
CT	Computed tomography
EASL	European Association for the Study of the Liver
HBP	Hepatobiliary phase
HCC	Hepatocellular carcinoma
KLCA-NCC	Korean Liver Cancer Association-National Cancer Center
LI-RADS	Liver Imaging Reporting and Data System
LT	Liver transplantation
LRT	Local regional therapy
MRI	Magnetic resonance imaging
MVI	Microvascular invasion
PET	Positron emission tomography
RECIST	Response evaluation criteria in solid tumors
TACE	Transarterial chemoembolization
TARE	Transarterial radioembolization
TIV	Tumor in vein
UCSF	University of California San Francisco
VETC	Vessel Encapsulated Tumor Clusters
WHO	World Health Organization
